# Vitamin K supplementation and bone mineral density in dialysis: results of the double-blind, randomized, placebo-controlled RenaKvit trial

**DOI:** 10.1093/ndt/gfac315

**Published:** 2022-12-02

**Authors:** Karin Levy-Schousboe, Peter Marckmann, Marie Frimodt-Møller, Christian D Peters, Krista D Kjærgaard, Jens D Jensen, Charlotte Strandhave, Hanne Sandstrøm, Mette F Hitz, Bente Langdahl, Peter Vestergaard, Claus L Brasen, Anne Schmedes, Jonna S Madsen, Niklas R Jørgensen, Jens B Frøkjær, Niels E Frandsen, Inge Petersen, Ditte Hansen

**Affiliations:** Department of Medicine, Zealand University Hospital, Roskilde, Denmark; Department of Medicine Sønderborg-Tønder, Hospital Sønderjylland, Sønderborg, Denmark; Steno Diabetes Center, Copenhagen, Denmark; Department of Nephrology, Herlev University Hospital, Copenhagen, Denmark; Department of Renal Medicine, Aarhus University Hospital, Aarhus, Denmark; Department of Renal Medicine, Aarhus University Hospital, Aarhus, Denmark; Department of Renal Medicine, Aarhus University Hospital, Aarhus, Denmark; Department of Nephrology, Aalborg University Hospital, Aalborg, Denmark; Department of Radiology, Zealand University Hospital, Roskilde, Denmark; Department of Medicine, Zealand University Hospital, Køge, Denmark; Department of Clinical Medicine, University of Copenhagen, Copenhagen, Denmark; Department of Endocrinology and Internal Medicine, Aarhus University Hospital, Aarhus, Denmark; Department of Clinical Medicine, Aarhus University, Aarhus, Denmark; Department of Endocrinology, Aalborg University Hospital, Aalborg, Denmark; Steno Diabetes Center North Denmark, Aalborg University Hospital, Aalborg, Denmark; Department of Clinical Medicine, Aalborg University, Aalborg, Denmark; Department of Biochemistry and Immunology, Lillebælt Hospital, University Hospital of Southern Denmark, Denmark; Department of Regional Health Research, Faculty of Health Sciences, University of Southern Denmark, Odense, Denmark; Department of Biochemistry and Immunology, Lillebælt Hospital, University Hospital of Southern Denmark, Denmark; Department of Biochemistry and Immunology, Lillebælt Hospital, University Hospital of Southern Denmark, Denmark; Department of Regional Health Research, Faculty of Health Sciences, University of Southern Denmark, Odense, Denmark; Department of Clinical Medicine, University of Copenhagen, Copenhagen, Denmark; Department of Clinical Biochemistry, Rigshospitalet, Copenhagen, Denmark; Department of Clinical Medicine, Aalborg University, Aalborg, Denmark; Department of Radiology, Aalborg University Hospital, Aalborg, Denmark; Department of Medicine, Zealand University Hospital, Roskilde, Denmark; Open Patient Data Explorative Network, Department of Clinical Research, University of Southern Denmark and Odense University Hospital, Odense, Denmark; Department of Nephrology, Herlev University Hospital, Copenhagen, Denmark; Department of Clinical Medicine, University of Copenhagen, Copenhagen, Denmark

**Keywords:** bone mineral density, chronic kidney disease, end-stage kidney disease, menaquinone-7, mineral and bone disorder

## Abstract

**Background:**

Vitamin K deficiency is highly prevalent in patients on dialysis and may contribute to their low bone mineral density (BMD) and increased risk of fracture. This study investigated the effect of menaquinone-7 (MK-7) supplementation on BMD in patients on chronic dialysis.

**Methods:**

In a multicentre, double-blind, placebo-controlled intervention trial, 123 patients on chronic dialysis were randomised to a daily oral supplement of either MK-7 360 µg or placebo for 2 years. BMD of the distal radius (1/3, mid, ultradistal and total), femoral neck, lumbar spine (L1–L4) and whole body was assessed by dual-energy X-ray absorptiometry. Serum levels of vitamin K1 and MK-7 and plasma levels of total osteocalcin, dephosphorylated-uncarboxylated matrix Gla protein and protein induced by vitamin K absence II were measured to assess vitamin K status.

**Results:**

After 2 years, an accelerated BMD loss of the 1/3 distal radius was found with MK-7 supplementation {mean difference of changes relative to placebo −0.023 g/cm^2^ [95% confidence interval (CI) −0.039 to −0.008]}, whereas the decrease in lumbar spine BMD seen in the placebo group was prevented [mean difference of changes between groups 0.050 g/cm^2^ (95% CI 0.015–0.085)]. No significant effects were observed at the remaining skeletal sites. Vitamin K status strongly improved in MK-7-supplemented participants.

**Conclusion:**

Compared with placebo, an accelerated BMD loss of the 1/3 distal radius was found after 2 years of MK-7 supplementation, whereas a decline in lumbar spine BMD was prevented. As such, MK-7 supplementation might modify BMD site-specifically in patients on dialysis. In aggregate, our findings do not support MK-7 supplementation to preserve bone in patients on dialysis.

## Graphical Abstract

**Graphical Abstract:**
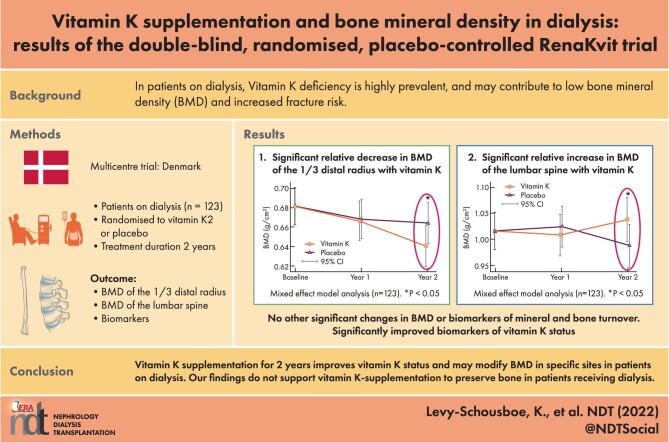

KEY LEARNING POINTS **What is already known about this subject?**

- In patients with chronic kidney disease on dialysis treatment, low bone mineral density (BMD) is prevalent.
- Low BMD is associated with an increased risk of bone fracture and mortality.
- Vitamin K deficiency may contribute to low BMD.

**What this study adds?**

- In a 2-year randomised, double-blind, placebo-controlled study of patients on chronic dialysis treatment, an accelerated BMD loss of the 1/3 distal radius but preserved BMD of the lumbar spine were seen with 2 years of MK-7 supplementation compared with the changes in BMD with placebo in an intention-to-treat analysis. BMD at the other sites measured was unaffected.
- Vitamin K supplementation improved the vitamin K status compared with placebo.

**What impact this may have on practice or policy?**

- The results of the present study indicate that MK-7 supplementation might modify BMD site-specifically in patients receiving dialysis treatment but, in aggregate, do not support MK-7 supplementation to preserve bone.

## INTRODUCTION

As kidney function declines, disturbances in the mineral metabolism cause abnormalities in bone turnover, mineralization and bone loss, as well as increased vascular and other soft-tissue calcification, the so-called chronic kidney disease–mineral and bone disorder (CKD-MBD) [1]. In patients with end-stage kidney disease (ESKD) on dialysis, the risk of fractures is increased up to 4 times compared with the general population [2]. Fractures are associated with increased morbidity and all-cause mortality [3]. Compared with the general population, low bone mineral density (BMD) is more frequent [4] and predicts future fractures in patients on dialysis treatment [5].

BMD may be affected by vitamin K status. In observational studies, vitamin K levels were positively associated with BMD in the elderly general population [6] and in patients on dialysis [7]. Furthermore, reduced levels of vitamin K are associated with an increased risk of fracture in both the elderly general population [8] and in patients on dialysis [9, 10]. In addition, treatment with vitamin K antagonists increases fracture risk [10].

Patients treated with dialysis are very often vitamin K deficient, with highly elevated levels of vitamin K–dependent proteins. Vitamin K supplementation has been demonstrated to improve vitamin K status, as systemic levels of osteocalcin [OC; as undercarboxylated osteocalcin (ucOC)], matrix Gla protein [MGP; as dephosphorylated-uncarboxylated matrix Gla protein (dp-ucMGP)] and protein induced by vitamin K absence II (PIVKA-II) significantly decreased during supplementation [11, 12]. So far, the effect of vitamin K supplementation on BMD in patients on dialysis treatment has not been reported by any studies.

The aim of this randomised, double-blind, placebo-controlled, study was to investigate the effect of vitamin K supplementation on BMD in patients on dialysis treatment.

## MATERIALS AND METHODS

### Study design

The RenaKvit trial was a 2-year randomised, multicentre, double-blind, placebo-controlled study examining the effects of vitamin K supplementation as menaquinone-7 (MK-7) on bone and vascular calcification in patients on dialysis treatment. The study design and the effects on vascular calcification have been described previously [13].

The study was approved by the Scientific Ethics Committee for the Region of Zealand (SJ-511) and the Danish Data Protection Agency and was registered at ClinicalTrial.gov (NCT02976246). The supporting CONSORT checklist can be found in Supplementary Table 1S.

### Study population and intervention

In brief, eligible participants were adult patients on chronic dialysis treatment not treated with vitamin K antagonists, recombinant parathyroid hormones, anti-osteoporotic drugs or vitamin K supplements. Participants were excluded during follow-up if they initiated vitamin K antagonist treatment, were <50% adherent to or had non-acceptable side effects from the study tablets or received a kidney transplant. Informed consent was obtained from all participants included in the study.

Participants were randomised to receive one daily tablet containing 360 µg MK-7 or a visually identical placebo tablet using concealed block randomisation with blocks of four (2:2). Each block consisted of patients treated with the same dialysis modality at the same dialysis centre. Double blinding was maintained until the last patient's last visit.

Kappa Bioscience AS, Oslo, Norway, produced the synthetic MK-7 (K2VITAL Delta), according to the Hazard Analysis Critical Control Point principles, and Orkla Care AS, Ishøj, Denmark, produced all tablets used in the study.

Adverse events (AEs) and serious AEs (SAEs) were monitored according to the procedure described in Supplementary Table 2S.

### Outcomes

The primary outcome was changes in BMD of the 1/3 distal radius, since BMD of the 1/3 distal radius predicts fractures in patients on dialysis treatment [5, 14, 15]. Secondary outcomes were changes in BMD of the remaining distal radial sites (mid, ultradistal and total), lumbar spine (L1–L4), femoral neck and whole body, as well as changes in biochemical markers of vitamin K status and bone metabolism, changes in abdominal aortic calcification (AAC) scores and clinical outcomes (bone fracture, parathyroidectomy, thromboembolic event and death) during the study period.

### Measurements

#### Dual-energy X-ray absorptiometry (DXA)

BMD of the distal half of the left radius [divided into the 1/3 (most proximal 20 mm), mid and ultradistal radius (most distal 15 mm)], lumbar spine (L1–L4), left femoral neck and whole body were assessed by DXA. DXA procedures are described in detail in Supplementary Material 4S.

#### Biochemical measurements

Serum vitamin K1 and MK-7 were analysed by mass spectrometry according to Boegh *et al.* [16] with modifications for MK-7 analysis as previously described [13]. Biochemical procedures are described in detail in Supplemental Material 4S.

#### Conventional X-ray of the lumbar spine

AAC scores were quantified as per Kauppila *et al.* [17] using a conventional laterally exposed X-ray of the lumbar spine (L1–L4). All X-rays were reviewed by the same experienced radiologist who was blinded to treatment allocation. The number of lumbar vertebral compression fractures (L1–L4), defined as a vertebral height reduction of at least 20% or 4 mm [18], was assessed at baseline and after 2 years of intervention.

### Statistical methods

No data on changes in BMD over time of the 1/3 distal radius were available in patients on dialysis when the study was planned. The sample size calculation was therefore based on cross-sectional data in which a difference in BMD of the 1/3 distal radius of 0.26 g/cm^2^ in patients on dialysis with or without fractures and an overall standard deviation (SD) in BMD of the 1/3 distal radius of 0.46 g/cm^2^ was found [19]. To detect a minimal relevant change in the 1/3 distal radius BMD of 0.26 g/cm^2^ (5% significance level, 80% power) at least 2 × 49 patients were needed. With an expected annual dropout rate of 20% in our study population with a well-described high mortality, we aimed at including at least 2 × 70 patients in the study. Parametric and non-parametric statistics were applied on normally and non-normally distributed data, respectively. Categorical data were analysed with the Pearson's chi-squared or Fisher's exact test. Mixed effects linear regression models were used to estimate longitudinal treatment effects. With the applied methodology, missing values were handled and list exclusion avoided, meaning that analyses were intention-to-treat (ITT) analyses. Maximum likelihood procedures were used for estimation, thus, under the assumption of missing at random, unbiased effect sizes are estimated. The analyses were adjusted for baseline measures by constraining the baseline means of the two study groups to be equal, thereby considering participants as a random sample of the background prior to intervention, as suggested by Twisk *et al.* [20]. Examination of the underlying model assumptions was performed by visual inspection of QQ-plots of residuals as well as residuals versus predicted plots. If deviation from the assumption of variance homogeneity of the residuals was observed, the analyses were modified allowing for distinct error variances.

Due to severe heteroscedasticity in the MK-7 group, the variance of residuals at follow-up measurements was allowed to differ from residual variances at baseline. All analyses were repeated with adjustments for age and sex (partly adjusted) and adjustments for sex, age and baseline measures of 25-hydroxyvitamin D and dp-ucMGP (fully adjusted). The testing of secondary endpoints was not hierarchical. Mixed effects models were implemented in Stata version 16 (StataCorp, College Station, TX, USA) and other data processing and analyses using SPSS version 26 (IBM, Armonk, NY, USA). *P*-values <.05 were considered statistically significant.

## RESULTS

**Figure 1:**
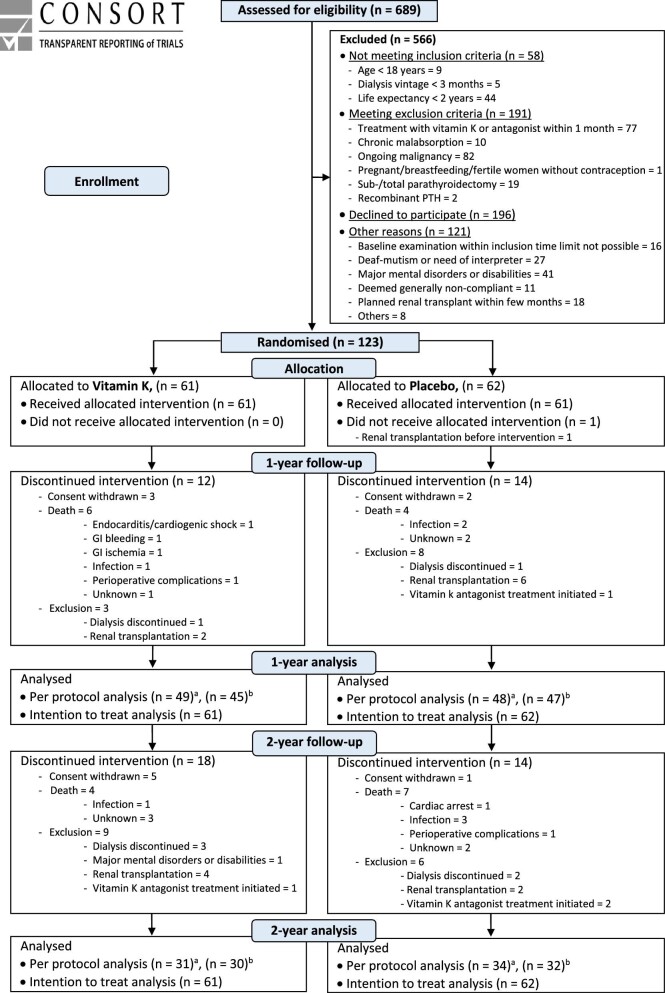
CONSORT flow diagram. GI, gastro intestinal; PTH, parathyroid hormone. ^a^Number of overall completing participants. ^b^Number of participants completing the 1/3 distal radius DXA (primary outcome).

A total of 689 patients on dialysis were screened for eligibility (Fig. 1). Of those, 123 patients were enrolled and randomised to either MK-7 (*n* = 61) or placebo (*n* = 62). Twenty-six participants (21%) dropped out during year 1, leaving 97 participants (79%) available for the 1-year follow-up. An additional 32 participants dropped out during year 2, leaving 65 participants (53%) available for the 2-year follow-up (Fig. 1). All participants were Caucasian except one participant of Asian ancestry and one of Latino ancestry. Baseline characteristics are shown in Tables 1 and 2.

**Table 1:** Baseline characteristics of participants.

	All participants randomised (*N* = 123)	
Characteristics	Vitamin K	Placebo
Participants, *n*	61	62
Males, *n* (%)	45 (74)	50 (81)
Age (years), mean ± SD	65.2 ± 11.1	63.7 ± 12.0
Height (cm), mean ± SD	172.9 ± 9.6	174.0 ± 8.6
Body mass index (kg/m^2^), mean ± SD	26.3 ± 5.1	27.6 ± 5.6
Smokers, *n* (%)		
Current	10 (17)	11 (18)
Former	31 (51)	21 (34)
Comorbidity, *n* (%)		
Previous fracture^a^	3 (5)	4 (6)
Cerebral stroke^b^	8 (13)	5 (8)
Peripheral arterial disease^c^	6 (10)	5 (8)
Ischemic heart disease^d^	7 (11)	4 (6)
Heart failure^e^	8 (13)	8 (13)
Aetiology to ESKD^f^, *n* (%)		
Polycystic kidney disease	9 (15)	7 (11)
Diabetic nephropathy	7 (11)	16 (26)
Glomerulonephritis	10 (16)	10 (16)
Vascular-hypertensive nephropathy	12 (20)	13 (21)
Post-renal nephropathy	1 (2)	0 (0)
Focal segmental glomerulosclerosis	1 (2)	0 (0)
Vasculitis	1 (2)	1 (2)
Chronic interstitial nephritis	0 (0)	1 (2)
Hereditary nephropathy	0 (0)	1 (2)
Other	11 (18)	6 (10)
Unknown	9 (15)	7 (11)
Dialysis modality, *n* (%)		
Haemodialysis	41 (67)	38 (61)
Peritoneal dialysis	20 (33)	21 (34)
Hybrid dialysis^g^	0 (0)	3 (5)
Dialysis vintage (months), median (IQR)	26.0 (9.3–46.0)	17.0 (8.8–42.3)
Former kidney transplant, *n* (%)	8 (13)	12 (19)
Medication, *n* (%)		
Vitamin D		
Native	40 (52)	37 (48)
Activated	40 (48)	44 (52)
Phosphate binders		
Calcium-containing	28 (42)	38 (58)
Non-calcium-containing	39 (46)	45 (54)
Calcimimetics (cinacalcet, etelcalcetid)	11 (44)	14 (56)
Biochemistry, median (IQR)		
S-vitamin K1 (nmol/l)	0.36 (0.21–0.66)	0.46 (0.28–0.65)
S-MK-7 (nmol/l)	0.40 (0.25–0.71)	0.49 (0.30–0.76)
P-tOC (ng/ml)	186.4 (112.4–349.9)	154.1 (69.7–228.4)
P-dp-ucMGP (pmol/l)^h^	1870 (1315–2651)	1941 (1449–2629)
P-PIVKA-II (ng/ml)^h^	36.1 (22.3–54.4)	25.6 (21.1–42.1)
P-25-OH-vitamin D (D2 + D3) (nmol/l), mean ± SD	74.2 ± 35.2	61.6 ± 28.7
S-1,25(OH)_2_ vitamin D (pmol/l), mean ± SD	54.4 ± 30.8	49.0 ± 25.7
P-iPTH (pmol/l)	28.5 (20.2–43.6)	23.7 (12.3–43.6)
P-FGF-23 (ng/l)	5 015 (1820–11 083)	3463 (698–12 530)
P-ionized calcium (mmol/l), mean ± SD	1.18 ± 0.09	1.19 ± 0.07
P-phosphate (mmol/l), mean ± SD	1.69 ± 0.41	1.59 ± 0.44
P-magnesium (mmol/l), mean ± SD	1.04 ± 0.17	1.00 ± 0.22
P-BSAP (µg/l)	10.7 (7.6–15.9)	10.6 (5.9–15.6)
P-CTX-1 (ng/l), mean ± SD	2086 ± 1228	1608 ± 965
P-P1NP (µg/l)	247.8 (135.6–423.1)	205.4 (113.9–394.1)
P-albumin (g/l), mean ± SD	34 ± 3.9	34 ± 4.4

B: blood; P: plasma; S: serum; FGF-23: fibroblast growth factor 23; BSAP: bone-specific alkaline phosphatase; CTX-1: type I collagen cross-linked C-telopeptide; P1NP, total procollagen type 1 N-terminal propeptide.

a Previous fracture of the vertebra, upper- or forearm and/or upper or lower leg within the last 5 years and verified by X-ray.

^b^Former sudden onset of focal neurological outcome >24 h duration, radiologically verified.

^c^Former amputation, peripheral by-pass operation, Fontaine 3rd degree (resting pain) or 4th degree (ischaemic ulcer/gangrene), ankle blood pressure <50 or toe blood pressure <40 mmHg.

d Former medically and biochemically verified acute myocardial infarction, percutaneous coronary intervention or coronary artery bypass graft.

^e^Ejection fraction <40% assessed by echocardiography.

^f^According to medical records.

^g^Hybrid dialysis treatment consisting of alternately haemo- and peritoneal dialysis.

^h^Normal reference range: dp-ucMGP 366–-646 pmol/l, PIVKA-II 8.4–131.0 ng/ml according to the manufacturer (Supplementary Material 4S).

**Table 2:** Baseline bone characteristics of participants assessed by DXA.

	All participants randomised	
Characteristics	Vitamin K	Placebo
Participants, *n*	61	62
1/3 distal radius		
BMD (g/cm^2^)	0.669 ± 0.099	0.695 ± 0.110
T-score	−2.08 ± 1.53	−1.82 ± 1.59
Z-score	−0.73 ± 1.61	−0.58 ± 1.58
Mid-distal radius		
BMD (g/cm^2^)	0.535 ± 0.096	0.560 ± 0.101
T-score	−2.68 ± 1.66	−2.25 ± 1.63
Z-score	−1.59 ± 1.77	−1.31 ± 1.59
Ultradistal radius		
BMD (g/cm^2^)	0.375 ± 0.800	0.389 ± 0.097
T-score	−2.34 ± 1.22	−2.19 ± 1.50
Z-score	−1.19 ± 1.37	−1.12 ± 1.59
Total distal radius		
BMD (g/cm^2^)	0.520 ± 0.089	0.543 ± 0.098
T-score	−2.55 ± 1.45	−2.30 ± 1.58
Z-score	−1.31 ± 1.60	−1.14 ± 1.62
Lumbar spine (L1-L4)		
BMD (g/cm^2^)	1.003 ± 0.202	1.030 ± 0.195
T-score	−0.63 ± 1.80	−0.49 ± 1.73
Z-score	0.34 ± 1.96	0.44 ± 1.80
Femoral neck		
BMD (g/cm^2^)	0.777 ± 0.145	0.797 ± 0.165
T-score	−1.57 ± 0.94	−1.41 ± 1.03
Z-score	−0.88 ± 0.91	−0.61 ± 1.05
Whole body		
BMD (g/cm^2^)	1.049 ± 0.107	1.059 ± 0.123
T-score	−1.43 ± 1.12	−1.44 ± 1.36
Z-score	−0.76 ± 1.02	−0.76 ± 1.21

Data presented as mean ± SD unless stated otherwise.

### DXA

DXA scans were performed in 92 of the 97 participants at year 1 and in 62 of the 65 participants completing the study at year 2 (Fig. 1). Causes of missing measurements are shown in Supplementary Table 5S. At baseline, the number of participants with a T-score below the osteoporosis defining threshold of −2.5 at any BMD site did not differ between the MK-7 and placebo groups (69% and 54%, respectively; *P* = .10).

#### Distal radius BMD

**Figure 2:**
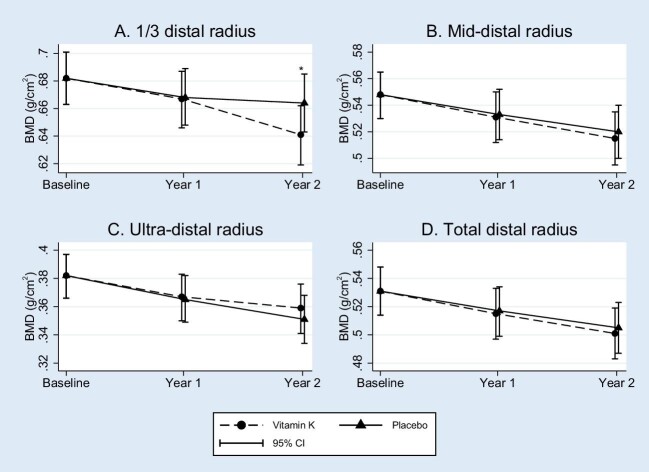
Effects of 2-years of supplementation with vitamin K2 (MK-7, 360 μg daily) or placebo: Bone mineral density of the distal radius. Mixed effect model analysis of changes in bone mineral density of the distal radius. (**A**) The 1/3 distal radius. (**B**) Mid-distal radius. (**C**) Ultra-distal radius. (**D**) Total distal radius (*n* = 120_baseline_, 92_year 1_ and 62_year 2_). Variables are unadjusted and presented as mean and 95% confidence intervals (CI). Significant between-group differences *: *P* ≤ .005. BMD; bone mineral density.

After 1 year, BMD of the 1/3 distal radius decreased significantly in both the group treated with MK-7 {−0.015 g/cm^2^ [95% confidence interval (CI) −0.025 to −0.005]} and placebo [−0.013 g/cm^2^ (95% CI −0.023 to −0.004)] with no between-group difference (*P* = .81) (Fig. 2A and Table 3). The absolute BMD levels were similar between groups (*P* = .13) (Supplementary Table 6S). After 2 years, BMD of the 1/3 distal radius was significantly decreased in both the MK-7 [−0.041 g/cm^2^ (95% CI −0.052 to −0.030)] and the placebo groups [−0.018 g/cm^2^ (−0.029 to −0.007)]. The decrease was significantly larger in the MK-7 group (*P* = .004) (Fig. 2A and Table 3) and the absolute level of BMD was significantly lower in the MK-7 group after 2 years (*P* = .03) (Supplementary Table 6S).

**Table 3:** Effects of 2 years of supplementation with vitamin K2 (MK-7, 360 µg/day) or placebo: Significant changes in bone mineral density.

		Unadjusted analyses				Unadjusted analyses		Partly adjusted analyses		Fully adjusted analyses	
		Mean levels (95% CI)		Mean change in study groups from baseline (95% CI)							
BMD (g/cm^2^)	Time	Vitamin K	Placebo	Vitamin K	Placebo	Differences of changes between study groups from baseline (95% CI)	*P*-value	Differences of changes between study groups from baseline (95% CI)	*P*-value	Differences of changes between study groups from baseline (95% CI)	*P*-value
1/3 distal radius	Baseline	0.682 (0.663–0.701) (*n* = 120)									
	Year 1	0.667 (0.646–0.687) (*n* = 45)	0.668 (0.648–0.689) (*n* = 47)	−0.015 (−0.025 to −0.005)	−0.013 (−0.023 to −0.004)	−0.002 (−0.015–0.012)	.81	−0.002 (−0.015–0.012)	.79	−0.001 (−0.015–0.012)	.86
	Year 2	0.641 (0.619–0.662) (*n* = 30)	0.664 (0.643–0.685) (*n* = 32)	−0.041 (−0.052 to −0.030)	−0.018 (−0.029 to −0.007)	−0.023 (−0.039 to −0.008)	.004	−0.023 (−0.039 to −0.008)	.004	−0.021 (−0.037 to −0.005)	.009
Lumbar spine (L1–L4)	Baseline	1.016 (0.980–1.052) (*n* = 119)									
	Year 1	1.008 (0.968–1.048) (*n* = 44)	1.025 (0.985–1.064) (*n* = 48)	−0.008 (−0.029–0.014)	0.009 (−0.012–0.029)	−0.017 (−0.046–0.013)	.27	−0.017 (−0.046–0.013)	.26	−0.019 (−0.049–0.011)	.23
	Year 2	1.038 (0.996–1.080) (*n* = 29)	0.988 (0.947–1.029) (*n* = 33)	0.022 (−0.003–0.047)	−0.028 (−0.052–−0.004)	0.050 (0.015–0.085)	.005	0.050 (0.015–0.084)	.005	0.050 (0.015–0.086)	.005
Femoral neck	Baseline	0.786 (0.759–0.814) (*n* = 118)									
	Year 1	0.766 (0.731–0.801) (*n* = 44)	0.775 (0.741–0.810) (*n* = 47)	−0.021 (−0.048–0.007)	−0.011 (−0.038–0.016)	−0.010 (−0.047–0.028)	.61	−0.008 (−0.046–0.029)	.66	−0.004 (−0.042–0.034)	.84
	Year 2	0.752 (0.712–0.791) (*n* = 28)	0.717 (0.679–0.755) (*n* = 32)	−0.035 (−0.068 to −0.002)	−0.069 (−0.101–−0.038)	0.035 (−0.010–0.079)	.13	0.037 (−0.008–0.081)	.12	0.043 (−0.003–0.088)	.07

Mixed effects model analysis of changes in BMD. Partly adjusted for gender and age; fully adjusted for baseline vitamin D and dp-ucMGP levels.

After both 1 and 2 years of intervention, BMD of the mid, ultradistal and total distal radius decreased significantly in both the MK-7 and placebo groups, but without significant differences in mean changes or absolute BMD levels between groups (all *P*-values >.05) (Fig. 2B–D and Supplementary Tables 6S and 7S).

Adjustments for gender and age, and additionally baseline vitamin D and vitamin K levels, did not modify the results of MK-7 supplementation on BMD in a stepwise model of any of the regions of the distal radius (Table 3 and Supplementary Table 7S).

#### Lumbar spine, femoral neck and whole body BMD

**Figure 3:**
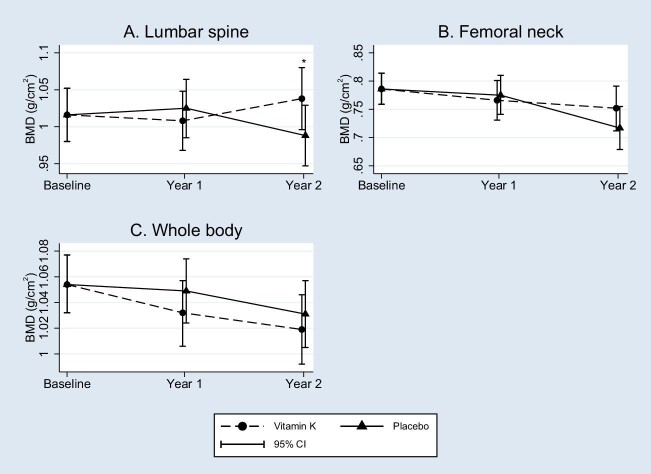
Effects of 2-years of supplementation with vitamin K2 (MK-7, 360 μg daily) or placebo: Bone mineral density. Mixed effect model analysis of changes in bone mineral density. (**A**) Lumbar spine (*n* = 119_baseline_, 92_year 1_ and 62_year 2_). (**B**) Femoral neck (*n* = 118_baseline_, 91_year 1_ and 60_year 2_). (**C**) Whole body (*n* = 105_baseline_, 80_year 1_ and 55_year 2_). Variables are unadjusted and presented as mean and 95% confidence intervals (CI). Significant between group differences *: *P* ≤ 0.005. BMD, bone mineral density.

After 1 year, BMD of the lumbar spine did not change significantly in either the MK-7 or placebo groups and the mean changes from baseline or absolute BMD levels did not differ between study groups (*P* = .27 and .53, respectively) (Fig. 3A, Table 3 and Supplementary Table 6S). After 2 years, lumbar spine BMD was maintained with MK-7 but decreased significantly with placebo, and changes differed significantly between study groups (*P* = .005). The absolute BMD levels did not differ significantly between study groups at year 2 (*P* = .70) (Fig. 3A, Table 3 and Supplementary Table 6S).

There were no significant differences between the study groups in BMD changes of the femoral neck or whole body (all *P*-values >.05), but after 2 years of MK-7 supplementation, femoral neck BMD was nearly maintained and changes between groups differed borderline significantly (*P* = .07) (Fig. 3B and C, Table 3 and Supplementary Tables 6S and 7S).

Adjustment for gender and age and baseline vitamin K and D levels did not significantly modify the results of MK-7 on BMD of the lumbar spine, femoral neck or whole body (Table 3 and Supplementary Table 7S).

### Biochemical outcomes

#### Markers of vitamin K

At baseline, participants were highly vitamin K deficient, as demonstrated by the elevated levels of plasma dp-uc-MGP (P-dp-uc-MGP) and PIVKA-II.

**Figure 4:**
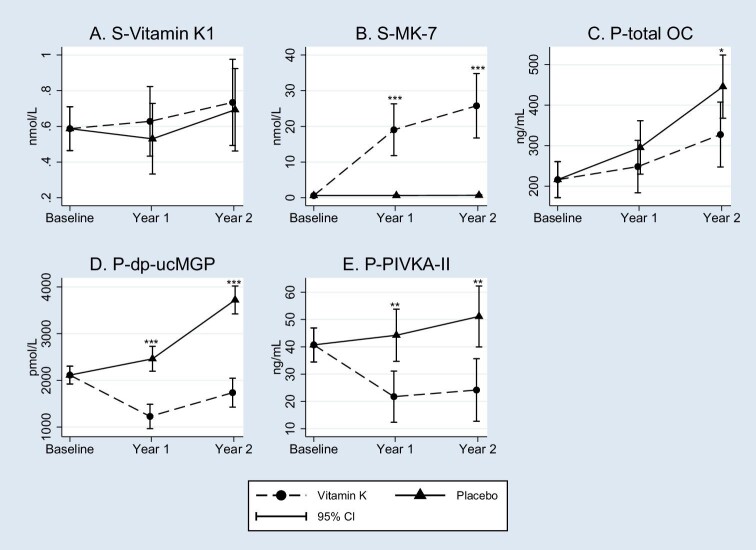
Effects of 2-year supplementation with vitamin K2 (MK-7, 360 μg daily) or placebo: Biochemical markers of vitamin K status. Mixed effect model analysis of changes in biochemical markers of vitamin K status. (**A**) vitamin K1 (*n* = 123_baseline_, 95_year 1_ and 65_year 2_). (**B**) MK-7 (*n* = 123_baseline_, 95_year 1_ and 65_year 2_). (**C**) total OC (*n* = 123_baseline_, 96_year 1_ and 60_year 2_). (**D**) dp-ucMGP (*n* = 123_baseline_, 96_year 1_ and 65_year 2_). (**E**) PIVKA-II (*n* = 122_baseline_, 96_year 1_ and 64_year 2_). Variables are unadjusted and presented as mean and 95% Confidence intervals (CI). Significant group differences (mean (95% CI)) at *P < .05, **P = 0.001, ***P < .001. dp-ucMGP, Dephosphorylated-uncarboxylated Matrix Gla Protein; MK-7, Menaquinone 7; P, Plasma; PIVKA-II, protein induced by vitamin K Absence-II; S, Serum.

Serum MK-7 (S-MK-7) increased significantly in the MK-7 group but remained stable in the placebo group. The mean changes from baseline and the absolute MK-7 levels differed highly significantly between study groups (all *P*-values <.001) (Fig. 4 and Supplementary Table 8S).

Plasma total osteocalcin (tOC) levels were unchanged in the MK-7 group but increased significantly in the placebo group after 1 year. Over the 2-year study period, levels increased significantly in both study groups, yet to a larger extent with placebo (*P* = .03).

P-dp-ucMGP and PIVKA-II levels decreased significantly in the MK-7 group and increased significantly in the placebo group, with significant differences in changes and absolute levels between groups (all *P*-values <.001) (Fig. 4 and Supplementary Table 8S).

#### Markers of mineral and bone turnover

There were no significant differences between the study groups in the markers of mineral metabolism or bone turnover at any time (all *P*-values >.05) (Supplementary Table 9S).

#### Conventional X-ray of the lumbar spine

After 1 and 2 years, AAC scores increased in both study groups, but the changes from baseline (*P* = .51 and .41) and the absolute scores (*P* = .19 and .68) did not differ between groups (Supplementary Table 10S).

The number and extent of lumbar vertebral compression fractures did not differ between the study groups at baseline (*P* = .16) and remained unchanged after 2 years (Supplementary Table 11S).

#### Clinical outcomes

The number of participants who experienced bone fracture, parathyroidectomy, a thromboembolic event or death during the study did not differ between study groups (ITT analyses; all *P*-values >.05) (Supplementary Table 12S). Due to more events in one placebo-supplemented participant, the number of thromboembolic events differed between the study groups (*P* = .02).

#### Adherence and AEs

The mean adherence to study tablets was >90% after year 1 and 2 in both study groups. A total of 110 participants experienced 968 AEs during the trial and 481 SAEs occurred in 90 participants.

The number of participants who experienced an AE or SAE did not differ between groups (all *P*-values >.05) (Supplementary Tables 2S and 3S).

## DISCUSSION

In the present randomised, double-blind, placebo-controlled trial, the effects on bone of daily supplementation with 360 µg vitamin K2 (MK-7) were compared with placebo in 123 patients receiving chronic dialysis treatment. In the ITT analysis, an accelerated BMD loss of the 1/3 distal radius was observed with MK-7 supplementation compared with placebo, whereas the decrease in lumbar spine BMD observed in the placebo-supplemented participants was prevented with MK-7. The RenaKvit trial is the first to investigate the effect of vitamin K supplementation on BMD in patients on dialysis treatment.

Surprisingly, we found the primary outcome, BMD of the 1/3 distal radius, to decrease ≈3% after 2 years of MK-7 supplementation compared with placebo, whereas BMD of the remaining distal radius sites was unaffected. However, these findings could not be confirmed in per protocol analysis. This may be because the participants able to complete the study represent only a subgroup of participants not randomly distributed between the groups. It may also be due to a type 1 statistical error in the ITT analysis. Four randomised studies, all in postmenopausal women with no kidney impairment, have examined the 2-year effect of vitamin K supplementation on distal radius BMD. One open-labelled study in 396 Japanese women examined the 1/3 distal radius and found a reduced BMD loss with supplements of vitamin K2 (MK-4) compared with no treatment [21]. Another open-label, non-placebo-controlled study found an increase in ultradistal radius BMD with vitamin K supplementation compared with vitamin D and calcium supplementation [22]. Yet, vitamin K supplementation did not affect the ultradistal and mid-distal radius BMD in two blinded and placebo-controlled studies [23, 24]. The present study is the first to find a worsening of BMD at any skeletal site during vitamin K supplementation compared with placebo. The difference in the 1/3 distal radius BMD between the study groups represents 20–25% of the SD, which translates into an ≈10% increased risk of fracture in patients with CKD according to Jamal *et al.* [15] and Prasad *et al.* [25].

BMD of the lumbar spine decreased in the placebo group to a similar extent as reported in a previous 2-year observational study in patients on dialysis [26]. This decrease was prevented, as BMD was maintained in the MK-7-supplemented group. The observed difference in changes in lumbar spine BMD between the study groups, corresponding to an ≈5% BMD increase with MK-7 supplementation, translates into a 10% reduction in vertebral fractures according to observations in CKD [15, 25].

In postmenopausal women without kidney impairment, the influence of vitamin K on lumbar spine BMD varies. Whereas three neither blinded nor placebo-controlled Japanese randomised 2-year studies [22, 27, 28] and one Dutch randomised, blinded, placebo-controlled 3-year study [29] found similar protective effects on lumbar spine BMD, two 3-year interventional studies in Caucasian postmenopausal women found no effect of MK-7 or MK-4 on lumbar BMD [30, 31].

BMD of the femoral neck decreased in both study groups after 2 years, although to a lesser extent with MK-7 supplementation and with borderline significance between treatments groups. Four randomised, controlled trials in elderly Caucasian populations without kidney impairment investigated the 3-year effect of vitamin K supplementation on BMD of the femoral neck. Two studies observed increased BMD with supplements of MK-7 [29] and vitamin K1 [32], yet two other studies found no effect with supplements of lower dosages of MK-4 [31] or vitamin K1 [33]. No short-term studies have found any effect of vitamin K1, MK-4 or MK-7 on BMD [23, 24, 34–37].

In ESKD, low BMD predicts fractures [5, 38]. Osteoporosis is diagnosed by DXA of the lumbar spine and hip, as in the general population, although skeletal sites with a higher content of cortical bone, such as the 1/3 distal radius, hypothetically should be superior in discriminating fracture risk due to the effects of secondary hyperparathyroidism [14].

Similar to our findings, MK-7 supplementation had contrasting effects on trabecular and cortical bone as assessed by high-resolution peripheral quantitative computed tomography in a 3-year study of Caucasian postmenopausal women [30, 36]. This dual effect of MK-7 on bone metabolism appears to be similar to that of parathyroid hormone (PTH), which increases cortical resorption and trabecular bone formation simultaneously [39, 40]. In the present study, levels of intact PTH (iPTH) did not differ between study groups; still an interaction between MK-7 and iPTH cannot be excluded.

Vitamin D regulates the expression of OC and appropriate vitamin D status may therefore be of importance for the effects of vitamin K supplementation on bone [41]. In the present study, participants were vitamin D replete and adjustments for vitamin D status did not change the results.

In accordance with other studies [32, 35, 36], we found a significant lesser increase in tOC with MK-7 supplementation compared with placebo, which may reflect carboxylation of ucOC, which enables its binding to hydroxyapatite in the bone matrix, as seen in human *in vitro* studies [42]. The observed effect on tOC may reflect an improvement in bone mineral maturation and bone structure with MK-7 supplementation.

Even though markers of bone turnover may be affected by ESKD [43], MK-7 did not appear to influence bone turnover in terms of changes in bone turnover markers in the present study, which is in accordance with observations in the elderly general population [32, 35, 36].

In uraemia, this absence of convincing beneficial effects of MK-7 supplementation on bone might be explained by the profound disturbances in vitamin K lipoprotein distribution and metabolism as recently discussed by Kaesler *et al.* [44]. Accordingly, it should be noted that even though we demonstrated highly significant effects on functional vitamin K status, e.g. levels of dp-ucMGP, it still did not normalize after 2 years of vitamin K2 supplementation.

This study has strengths and limitations. Being a randomised, placebo-controlled, double-blind study, important biases were eliminated. Also, the high adherence to study supplementation demonstrated both by tablet counting and blood concentrations of MK-7 and PIVKA-II was an important strength.

According to our power calculation, we aimed at including 2 × 70 participants. Due to challenged recruitment, unfortunately we ended up with only 61 + 62 participants. Furthermore, the dropout rate in year 2 was higher than anticipated. For these reasons, only 30 + 32 patients contributed with 2-year primary outcome data, a number somewhat lower than required according to the power calculations (2 × 49). Nonetheless, we managed to detect significant changes in the primary outcome variable. It is possible that a larger sample size and a longer trial duration would have demonstrated more convincing differences in the response between treatment groups, and even other significant differences in outcomes as well.

Previous studies have reported worsening of BMD on the arteriovenous fistula arm [14]. The number of functioning fistulas was similar in the two study groups; unfortunately, neither the placement nor the number of closed fistulas was registered.

Lumbar spine BMD may be increased by osteoarthritis, compression fractures, severe aortic calcification, which is common in the dialysis population, and intestinal deposition of phosphate binders [45, 46]. In the present study, we cannot exclude that lumbar spine osteoarthritis may have been unevenly distributed in the two study groups. However, neither AAC scores nor vertebral fractures differed between the study groups. Although DXAs were performed with abdomens empty for dialysis fluids, intake of phosphate binders and leftover peritoneal dialysis fluids may have affected measurements of lumbar spine BMD. However, the number of participants treated with peritoneal dialysis and phosphate binders did not differ between study groups.

Finally, we cannot exclude a more pronounced effect if a higher dosage of MK-7 had been used, if vitamin K1 had been supplemented [44] or if only patients with a high risk of bone fracture such as female patients on dialysis with low BMD, a history of bone fracture or severe renal osteodystrophy were included.

In conclusion, 2 years of vitamin K (MK-7) supplementation may modify BMD site-specifically in patients receiving dialysis treatment, in addition to improvement in functional vitamin K status. Still, our findings do not support MK-7 supplementation to preserve bone in patients with ESKD receiving dialysis treatment.

## Supplementary Material

gfac315_Supplemental_File

## Data Availability

Our data sharing statement is available as Supplementary Table 13S.
