# Supplementary material for: Vitamin K supplementation and bone mineral density in dialysis: results of the double-blind, randomized, placebo-controlled RenaKvit trial
Source: Nephrol Dial Transplant. 2022 Dec 2;38(10):2131–42. doi: 10.1093/ndt/gfac315 (PMC10539208; doi:10.1093/ndt/gfac315)
Supplement: gfac315_Supplemental_File [file gfac315_supplemental_file.docx]

**Supplementary Materials: Table of Contents**

**Supplemental Table 1S**: The supporting CONSORT checklist.

**Supplemental Tables 2S + 3S**: Effects of 2-years of supplementation with vitamin K2 (MK-7, 360 µg daily) or placebo: Adverse events.

**Supplemental Material 4S:** Trial Measurements

**Supplemental Table 5S**: Effects of 2-years of supplementation with vitamin K2 (MK-7, 360 µg daily) or placebo: Completing participants with missing DXA scans.

**Supplemental Table 6S**: Effects of 2-years of supplementation with vitamin K2 (MK-7, 360 µg daily) or placebo: Bone mineral density in completing participants.

**Supplemental Table 7S:** Effects of 2-years of supplementation with vitamin K2 (MK-7, 360 µg daily) or placebo: Bone mineral density.

**Supplemental Table 8S:** Effects of 2-year supplementation with vitamin K2 (MK-7, 360 µg daily) or placebo: Biochemical markers of vitamin K status

**Supplemental Table 9S:** Effects of 2-year supplementation with vitamin K2 (MK-7, 360 µg daily) or placebo: Biochemical markers of mineral and bone turnover.

**Supplemental Table 10S:** Effects of 2-years of supplementation with vitamin K2 (MK-7, 360 µg daily) or placebo: Abdominal Aortic Calcification (AAC) scores.

**Supplemental Table 11S:** Effects of 2-years of supplementation with vitamin K2 (MK-7, 360 µg daily) or placebo: Lumbar vertebral fractures.

**Supplemental Table 12S:** Effects of 2-years of supplementation with vitamin K2 (MK-7, 360 µg daily) or placebo: Clinical outcomes.

**Supplemental Table 13S**: Data Sharing Statement.

**Supplemental Table 1S**. The supporting CONSORT checklist.


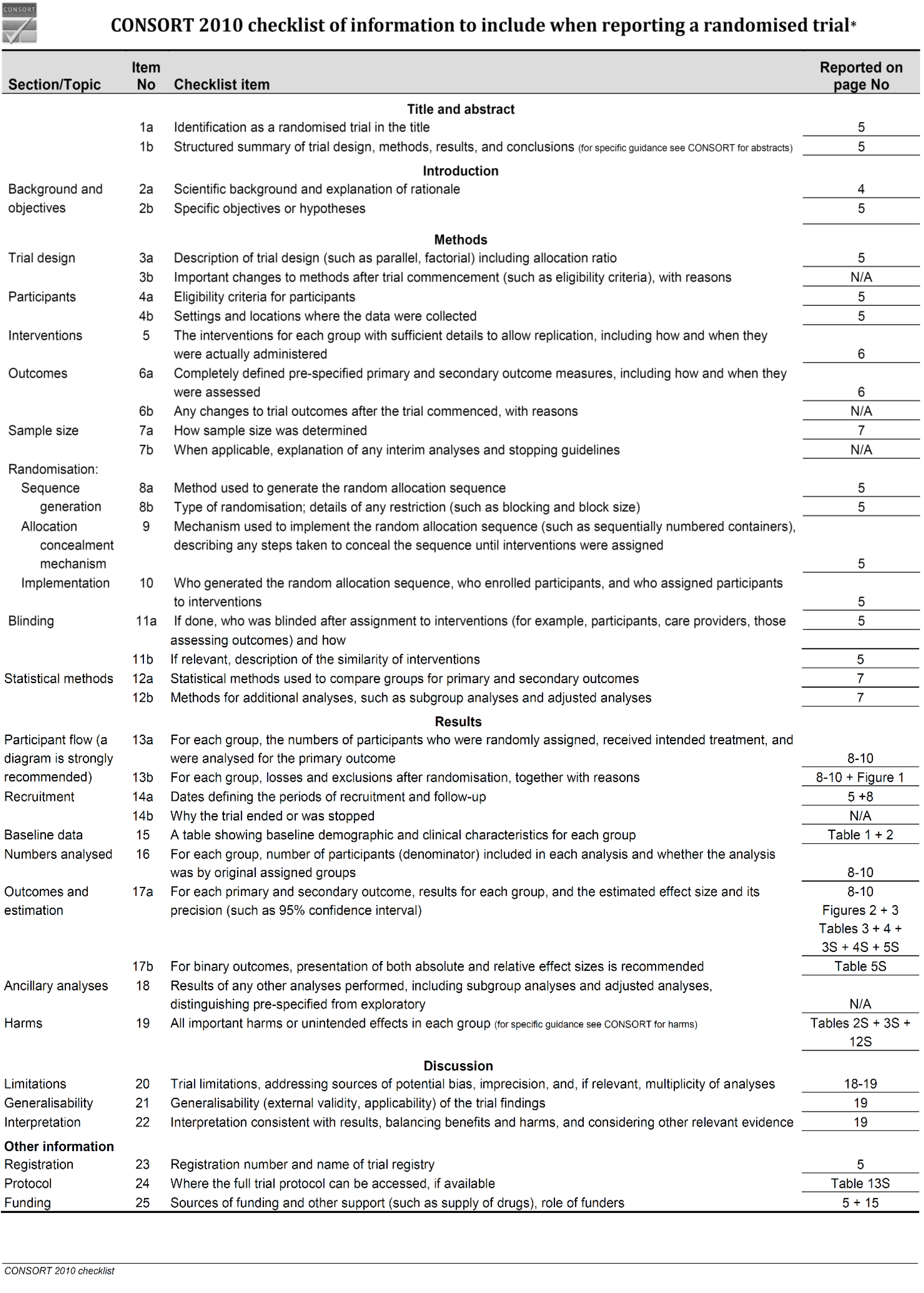


**Supplemental Table 2S.** Effects of 2-years of supplementation with vitamin K2 (MK-7, 360 µg daily) or placebo: Adverse events.

|  | **Vitamin K**  (n = 61) | | **Placebo**  (n = 62) | | **P**  **between study groups**^a^ | | **P**  **between study groups**^a^ | |
| --- | --- | --- | --- | --- | --- | --- | --- | --- |
|  | **AE** | **SAE** | **AE** | **SAE** | | **AE** | **SAE** |  |
| **Total** | 56 (447) | 47 (227) | 54 (521) | 43 (254) | | 0.40 | 0.34 |  |
| **Deaths** | 10 (10) | 10 (10) | 11 (11) | 11 (11) | | 0.85 | 0.85 |  |
| **Dialysis access related** | 27 (89) | 20 (56) | 34 (103) | 22 (64) | | 0.24 | 0.75 |  |
| Infectious | 16 (32) | 9 (19) | 17 (28) | 11 (14) | | 0.88 | 0.65 |  |
| Malfunction/-formation | 13 (32) | 7 (19) | 27 (47) | 12 (38) | | 0.08 | 0.23 |  |
| Stenosis | 6 (12) | 6 (10) | 4 (8) | 2 (4) | | 0.53 | 0.16 |  |
| Thrombosis | 6 (8) | 3 (8) | 9 (20) | 8 (18) | | 0.43 | 0.21 |  |
| **Cardiovascular/stroke** | 20 (44) | 12 (21) | 21 (54) | 15 (39) | | 0.90 | 0.55 |  |
| **Dermatologic** | 12 (21) | 1 (1) | 11 (22) | 2 (4) | | 0.78 | 1.00 |  |
| **Endocrinologic** | 8 (18) | 6 (7) | 7 (16) | 3 (9) | | 0.76 | 0.33 |  |
| **Fatigue** | 9 (13) | 8 (9) | 11 (15) | 10 (11) | | 0.65 | 0.64 |  |
| **Gastrointestinal** | 25 (42) | 15 (23) | 20 (44) | 12 (20) | | 0.32 | 0.48 |  |
| **Infectious** | 32 (92) | 21 (54) | 36 (84) | 20 (44) | | 0.53 | 0.80 |  |
| **Musculoskeletal** | 12 (27) | 4 (9) | 20 (75) | 6 (11) | | 0.11 | 0.75 |  |
| **Neurologic** | 7 (12) | 5 (5) | 8 (10) | 4 (5) | | 0.81 | 0.74 |  |
| **Oncologic** | 1 (1) | 0 (0) | 4 (5) | 2 (2) | | 0.37 | 0.50 |  |
| **Ophthalmologic** | 2 (8) | 0 (0) | 9 (16) | 1 (1) | | **0.03** | 1.00 |  |
| **Otorhinologic** | 7 (7) | 0 (0) | 3 (3) | 1 (1) | | 0.21 | 1.00 |  |
| **Psychiatric** | 2 (3) | 0 (0) | 6 (14) | 3 (5) | | 0.27 | 0.24 |  |
| **Respiratory** | 10 (17) | 6 (10) | 15 (37) | 7 (14) | | 0.28 | 0.79 |  |
| **Trauma^b^** | 8 (11) | 2 (4) | 14 (18) | 7 (9) | | 0.17 | 0.16 |  |
| **Urogenital** | 10 (29) | 8 (17) | 9 (13) | 3 (3) | | 0.77 | 0.13 |  |

**Supplemental Table 2S.** Variables are presented as number of participants experiencing an event (total number of events). ^a^ Fisher’s Exact test (n < 5), Pearson’s Chi-Squared test. ^b^ Trauma defined as any physical accident with or without accompanying fracture (see Table 6S and 10S).

Adverse events (AEs) were registered after 12 and 25 weeks, after 1 year, 78 weeks and by end of study after 2 years based on participants records and laboratory data. Furthermore, participants were asked for new complaints at visits. The following events are common in patients on chronic dialysis and were not considered AEs or serious AEs (SAEs): Changes in hemoglobin, unless caused by bleeding, hyperkalemia, abnormal plasma parathyroid hormone, phosphate, and calcium, unless requiring acute treatment or hospitalisation, metabolic acidosis and finally events related to dialysis treatment indifferent in type and severity as before the trial.

AE and SAE were registered after 12 and 25 weeks, after 1 year, 78 weeks and by end of study based on hospital records, laboratory data and participant-interviews at visits.

AE were classified as serious adverse event (SAE) according to the International Council for Harmonization (ICH) guideline for Good Clinical Practice (GCP). SAEs were reported to sponsor-investigator within 48 hours. The investigator classified the SAE as related to the study medication or not.

**Supplemental Table 3S.** Effects of 2-years of supplementation with vitamin K2 (MK-7, 360 µg daily) or placebo: AEs and SAEs possibly related to study supplement.

|  | **Vitamin K**  (n = 61) | | **Placebo**  (n = 62) | | **P**  **between study groups**^a^ | **P**  **between study groups**^a^ |
| --- | --- | --- | --- | --- | --- | --- |
|  | **AE** | **SAE** | **AE** | **SAE** | **AE** | **SAE** |
| **Total** | 16 (38) | 12 (29) | 18 (47) | 15 (36) | 0.73 | 0.55 |
| **Deaths** | 6 (6) | 6 (6) | 4 (4) | 4 (4) | 0.53 | 0.33 |
| **Access related**^b^ | 10 (21) | 6 (11) | 9 (21) | 7 (17) | 0.77 | 0.79 |
| Malfunction/-formation | 1 (2) | 1 (2) | 1 (2) | 1 (2) | 1.00 | 1.00 |
| Stenosis | 5 (7) | 4 (6) | 3 (5) | 1 (2) | 0.49 | 0.21 |
| Thrombosis | 6 (10) | 3 (8) | 5 (14) | 5 (13) | 0.73 | 0.72 |
| Decreased blood flow | 0 (0) | 0 (0) | 1 (1) | 0 (0) | 1.00 | 1.00 |
| **Central stenosis**^c^ | 3 (4) | 1 (1) | 2 (3) | 2 (2) | 0.68 | 1.00 |
| **Coronary stenosis**^d^ | 1 (1) | 1 (1) | 2 (2) | 2 (2) | 1.00 | 1.00 |
| **Peripheral stenosis**^e^ | 0 (0) | 0 (0) | 2 (3) | 2 (2) | 0.50 | 0.50 |
| **Thromboembolism**^f^ | 3 (3) | 2 (2) | 5 (7) | 5 (6) | 0.72 | 0.44 |

**Supplemental Table 3S.** Arterial events possibly related to MK-7 supplementation. Variables are presented as number of participants experiencing an event (total number of events). ^a^ Fisher’s Exact test (n < 5), Pearson’s Chi-Squared test; ^b^ Related to hemodialysis (dialysis filter, arteriovenous fistula or catheter); ^c^ Arterial stenosis proximal to the brachial and femoral arteries; ^d^ Coronary arterial stenosis not causing acute coronary syndrome, detected by coronary arteriography, e.g. in preparation for kidney transplantation; ^e^ Arterial stenosis distal to the subclavian and external iliac arteries, not related to access; ^f^ Arterial thromboembolism, not related to access or coronary arteries.

**Supplemental Material 4S.** Trial Measurements

*Dual-energy X-ray absorptiometry*

Dual-energy X-ray absorptiometry (DXA) was assessed using scanners Hologic Discovery or Hologic Horizon, Marlborough, MA, USA, with coefficients of variation (CV) of 1-2% at the investigated skeletal sites. The same DXA-scanner was used for each participant throughout the study. Participants on peritoneal dialysis treatment were examined without intraabdominal peritoneal dialysis fluid to minimize blurring of the lumbar spine. Technicians performing DXA scans were not aware of treatment allocation.

*Biochemical measurements*

Venous blood samples were collected with minimal stasis in evacuated blood collection tubes and were performed between 7.30 and 12 noon after an over-night fast. For standard serum (S-), the blood was allowed to clot at room temperature for 30 minutes prior to centrifugation at 3000 g for 10 min., whereas plasma (P-) analyses were centrifuged at 3000 g for 10 minutes either immediately (dephosphorylated-uncarboxylated MGP (dp-ucMGP), total-OC (tOC), Fibroblast Growth Factor 23 (FGF-23), PIVKA-II) or within 2 hours (Collagen 1 cross-linked C-terminal telopeptide fragments (CTX-1), Procollagen Type 1 N-Terminal Peptide (P1NP), Bone-Specific Alkaline Phosphatase (BSAP)). Samples were aliquoted in 500 μl Sarstedt polypropylene tubes and stored at −80°C until analysis. Technicians were not aware of treatment allocation.

S-vitamin K1 and MK-7 were analysed by mass spectrometry according to Boegh *et al.* [16] with modifications for MK-7 analysis as previously described [13]. This in-house developed method is very sensitive; the limits of quantification were 0.05 nmol/L and 0.50 nmol/L, and the intermediary precisions < 9% and 12%, respectively. All samples for vitamin K analyses were protected from sunlight at all times.

PIVKA-II, CTX-1 and P1NP were analysed in plasma using commercially available electrochemiluminescence immunoassays (ECLIA), according to the manufacturer’s instructions (Cobas e602 analyser, Roche Diagnostics, Denmark). The intermediary precision was <10%.

The isoform of dp-ucMGP, tOC and BSAP were analysed in plasma using commercially available ECLIA assays, according to the manufacturer’s instructions (ImmunoDiagnostic Systems Holdings PLC, East Boldon, United Kingdom). The intermediary precision was <10%. FGF-23 was determined in EDTA plasma using a chemiluminescence immunoassay assay (CLIA) on the automated analyser Liaison XL (Diasorin, Saluggia, Italy). 1,25-(OH)2 vitamin D was measured in serum using a CLIA in the IDS-iSYS automated analyser (ImmunoDiagnostic Systems Holdings PLC, East Boldon, United Kingdom). Intermediary precision for the two assays were <10% for P-FGF23 and <17% for S-1,25-(OH)2 vitamin D. Routine blood analyses (25-(OH)2 vitamin D, intact parathyroid hormone (iPTH), ionized calcium, phosphate, magnesium, albumin in plasma, and hemoglobin in whole blood) were performed at local certified laboratories with aligned reference intervals.

**Supplemental Table 5S.** Effects of 2-years of supplementation with vitamin K2 (MK-7, 360 µg daily) or placebo: Completing participants with missing DXA scans.

| **Overall completing** | | **(n)** |  | | | |  |  |
| --- | --- | --- | --- | --- | --- | --- | --- | --- |
|  | **97** | **Year 1** | | **65** | | **Year 2** | | |
| **Distal radius** | **92** | **DXA declined by patient = 3**  **Not performed = 2**  Death = 1 ^a^  Wrist fracture in cast = 1 | | **62** | | **DXA declined by patient = 1**  **Missing value = 1**  **Not performed = 1**  Cancellation (Danish Corona lock-down) = 1 | | |
| **Lumbar spine** | **92** | **DXA declined by patient = 3**  **Excluded = 1**  Artefact = 1  **Not performed = 1**  Death = 1 ^a^ | | **62** | | **DXA declined by patient = 1**  **Not performed = 2**  Cancellation (Danish Corona lock-down) = 1  Positioning prevented by severe kyphosis = 1 | | |
| **Femoral neck** | **91** | **DXA declined by patient = 3**  **Excluded = 1**  Total hip alloplastic = 1  **Missing value = 1**  **Not performed = 1**  Death = 1 ^a^ | | **60** | | **DXA declined by patient = 1**  **Excluded = 1**  Total hip alloplastic = 1  **Missing value = 1**  **Not performed = 2**  Cancellation (Danish Corona lock-down) = 1  Positioning prevented by severe kyphosis = 1 | | |
| **Whole body** | **80** | **DXA declined by patient = 2**  **Excluded = 1**  Leg alloplastic = 1  **Not performed = 14**  Death = 1 ^a^  Not performed at study center = 12  Severe vertigo developed = 1 | | | **55** | **DXA declined by patient = 1**  **Not performed = 8**  Severe vertigo developed = 1,  Positioning prevented by severe kyphosis = 1  Not performed at study center = 6  **Missing value = 1** | | |

**Supplemental Table 5S.** ^a^ Participated in all other 1-year analyses but died before the scheduled DXA scan.

**Supplemental Table 6S.** Effects of 2-years of supplementation with vitamin K2 (MK-7, 360 µg daily) or placebo:

Bone mineral density in completing participants.

| **BMD (g/cm2)** | **Baseline** | **Follow-up** | **P**  **change in study group^a^** | **P**  **between study groups follow-up^b^** | | **Change in study group** | **P**  **change between study groups^b^** | |
| --- | --- | --- | --- | --- | --- | --- | --- | --- |
| **1/3 distal radius** | | | | | | | | |
| **Completing year 1** Vitamin K; n= 45  Placebo; n= 47 | 0.664 (±0.106)  0.698 (±0.101) | 0.650 (±0.110)  0.684 (±0.104) | **< 0.001**  **< 0.001** | 0.13 | -0.014 (±0.022)  -0.014 (±0.025) | | | 0.99 |
| **Completing year 2**  Vitamin K; n= 30  Placebo; n= 32 | 0.649 (±0.112)  0.697 (±0.111) | 0.610 (±0.118)  0.678 (±0.122) | **< 0.001**  **0.02** | **0.03** | -0.039 (±0.050)  -0.018 (±0.040) | | | 0.07 |
| **Mid radius** | | | | | | | | |
| **Completing year 1** Vitamin K; n= 45  Placebo; n= 47 | 0.534 (±0.102)  0.562 (±0.094) | 0.518 (±0.102)  0.547 (±0.095) | **< 0.001**  **0.001** | 0.15 | -0.016 (±0.025)  -0.015 (±0.029) | | | 0.84 |
| **Completing year 2**  Vitamin K; n= 30  Placebo; n= 32 | 0.516 (±0.109)  0.567 (±0.106) | 0.485 (±0.112)  0.538 (±0.106) | **< 0.001**  **< 0.001** | 0.06 | -0.030 (±0.042)  -0.029 (±0.031) | | | 0.87 |
| **Ultra-distal radius** | | | | | | | | |
| **Completing year 1** Vitamin K; n= 45  Placebo; n= 47 | 0.372 (±0.087)  0.388 (± 0.092) | 0.357 (±0.085)  0.371 (±0.088) | **< 0.001**  **< 0.001** | 0.48 | -0.015 (±0.020)  -0.017 (±0.025) | | | 0.66 |
| **Completing year 2**  Vitamin K; n= 30  Placebo; n= 32 | 0.353 (± 0.086)  0.387 (±0.094) | 0.332 (±0.090)  0.356 (±0.092) | **< 0.001**  **< 0.001** | 0.31 | -0.022 (±0.029)  -0.031 (±0.037) | | | 0.26 |
| **Total distal radius** | | | | | | | | |
| **Completing year 1** Vitamin K; n= 45  Placebo; n= 47 | 0.518 (±0.095)  0.544 (±0.091) | 0.502 (±0.095)  0.529 (±0.092) | **< 0.001**  **< 0.001** | 0.17 | -0.016 (±0.019)  -0.015 (±0.019) | | | 0.87 |
| **Completing year 2**  Vitamin K; n= 30  Placebo; n= 32 | 0.500 (±0.101)  0.546 (±0.100) | 0.472 (±0.105)  0.519 (±0.101) | **< 0.001**  **< 0.001** | 0.08 | -0.029 (±0.037)  -0.027 (±0.027) | | | 0.81 |
| **Lumbar spine (L1-L4)** | | | | | | | | |
| **Completing year 1** Vitamin K; n= 44  Placebo; n= 48 | 1.013 (±0.203)  1.032 (±0.193) | 1.006 (±0.202)  1.025 (±0.200) | 0.43  0.31 | 0.53 | -0.007 (±0.057)  -0.007 (±0.046) | | | 0.99 |
| **Completing year 2**  Vitamin K; n= 29  Placebo; n= 33 | 0.980 (±0.191)  1.014 (±0.208) | 1.007 (±0.194)  0.980 (±0.212) | 0.14  **0.003** | 0.70 | 0.027 (±0.093)  -0.034 (±0.059) | | | **0.003** |
| **Femoral neck** | | | | | | | | |
| **Completing year 1** Vitamin K; n= 44  Placebo; n= 47 | 0.778 (±0.139)  0.798 (±0.160) | 0.755 (±0.136)  0.780 (±0.160) | **< 0.001**  **< 0.001** | 0.57 | -0.023 (±0.038)  -0.017 (±0.030) | | | 0.44 |
| **Completing year 2**  Vitamin K; n= 28  Placebo; n= 32 | 0.757 (±0.129)  0.795 (±0.164) | 0.730 (±0.115)  0.783 (±0.177) | 0.21  **0.004** | 0.83 | -0.026 (±0.108)  -0.071 (±0.130) | | | 0.15 |
| **Whole body** | | | | | | | | |
| **Completing year 1** Vitamin K; n= 37  Placebo; n= 43 | 1.058 (±0.111)  1.068 (±0.120) | 1.036 (±0.120)  1.059 (±0.127) | **0.001**  0.21 | 0.41 | -0.023 (±0.039)  -0.010 (±0.049) | | | 0.20 |
| **Completing year 2**  Vitamin K; n= 25  Placebo; n= 30 | 1.033 (±0.083)  1.062 (±0.138) | 0.998 (±0.100)  1.034 (±0.143) | **0.003**  **0.001** | 0.26 | -0.035 (±0.055)  -0.027 (±0.039) | | | 0.53 |

**Supplemental Table 6S.** Variables are presented as mean and standard deviation (X (±SD)). Abbreviations: DXA, Dual-energy X-ray absorptiometry; MK-7, menaquinone 7; BMD, bone mineral density. ^a^ Paired Student’s T-test; ^b^ Unpaired Student’s T-test.

**Supplemental Table 7S.** Effects of 2-years of supplementation with vitamin K2 (MK-7, 360 µg daily) or placebo: Additional changes in bone mineral density.

|  | | **Unadjusted analyses** | | | | | **Unadjusted analyses** | | | **Partly adjusted analyses** | | | **Fully adjusted analyses** | | |
| --- | --- | --- | --- | --- | --- | --- | --- | --- | --- | --- | --- | --- | --- | --- | --- |
| **BMD (g/cm^2^)** | **Time** | **Mean levels (95% CI)** | | | **Mean change in study groups from baseline (95% CI)** | | **Differences of changes between study groups from baseline (95% CI)** | | **P** | **Differences of changes between study groups from baseline (95% CI)** | | **P** | **Differences of changes between study groups from baseline (95% CI)** | | **P** |
| **Mid-distal radius** | Baseline | 0.548 (0.530;0.565)  (n=120) | | |  |  |  |  | |  |  | |  |  | |
|  | Year 1 | 0.531  (0.512;0.550) (n=45) | 0.533  (0.514;0.552)  (n=47) | | -0.017  (-0.026;-0.008) | -0.015  (-0.023;-0.006) | -0.002  (-0.014;0.010) | 0.69 | | -0.002  (-0.014;0.010) | 0.69 | | -0.002  (-0.014;0.010) | 0.75 | |
|  | Year 2 | 0.515  (0.495;0.535)  (n=30) | 0.520  (0.500;0.540)  (n=32) | | -0.033  (-0.043;-0.022) | -0.028  (-0.037;-0.018) | -0.005  (-0.019;0.009) | 0.48 | | -0.005  (-0.019;0.009) | 0.48 | | -0.003  (-0.018;0.011) | 0.64 | |
| **Ultra-distal radius** | Baseline | 0.382 (0.366;0.397)  (n=120) | | |  |  |  |  | |  |  | |  |  | |
|  | Year 1 | 0.367  (0.350;0.383) (n=45) | 0.365  (0.349;0.382)  (n=47) | | -0.015  (-0.023;-0.007) | -0.016  (-0.024;-0.009) | 0.001  (-0.009;0.012) | 0.79 | | 0.002  (-0.009;0.012) | 0.77 | | 0.003  (-0.008;0.014) | 0.56 | |
|  | Year 2 | 0.359  (0.341;0.376)  (n=30) | 0.351  (0.334;0.368)  (n=32) | | -0.023  (-0.032;-0.014) | -0.031  (-0.040;-0.022) | 0.008  (-0.005;0.020) | 0.22 | | 0.008  (-0.004;0.020) | 0.21 | | 0.008  (-0.004;0.021) | 0.20 | |
| **Total distal radius** | Baseline | 0.531 (0.514;0.548)  (n=120) | | |  |  |  |  | |  |  | |  |  | |
|  | Year 1 | 0.515  (0.497;0.533)  (n=45) | 0.517  (0.499;0.534)  (n=47) | | -0.016  (-0.023;-0.009) | -0.015  (-0.022;-0.008) | -0.002  (-0.011;0.008) | 0.76 | | -0.002  (-0.011;0.008) | 0.76 | | -0.001  (-0.011;0.009) | 0.86 | |
|  | Year 2 | 0.501  (0.483;0.519)  (n=30) | 0.505  (0.487;0.523)  (n=32) | | -0.030  (-0.038;-0.022) | -0.026  (-0.034;-0.018) | -0.004  (-0.016;0.007) | 0.49 | | -0.004  (-0.016;0.007) | 0.48 | | -0.003  (-0.015;0.009) | 0.64 | |
| **Whole body** | Baseline | 1.054 (1.032;1.077)  (n=105) | | |  |  |  |  | |  | | |  |  | |
|  | Year 1 | 1.032  (1.006;1.057)  (n=37) | | 1.049 1.024;1.074) (n=43) | -0.022  (-0.037;-0.008) | -0.005  (-0.019;0.008) | -0.017  (-0.037;0.003) | 0.09 | | -0.016  (-0.036;0.003) | 0.10 | | -0.018  (-0.038;0.002) | 0.08 | |
|  | Year 2 | 1.019  (0.992;1.046) (n=25) | | 1.031 (1.005;1.057) (n=30) | -0.036  (-0.053;-0.019) | -0.023  (-0.039;-0.008) | -0.012  (-0.035;0.011) | 0.29 | | -0.012  (-0.035;0.011) | 0.31 | | -0.013  (-0.036;0.010) | 0.28 | |

**Supplemental Table 7S.** Mixed effect model analysis of changes in BMD. Partly adjusted, adjustments for gender and age; Fully adjusted, additionally adjustments for baseline vitamin D and dp-ucMGP levels. Abbreviations: BMD, bone mineral density; CI, Confidence interval.

**Supplemental Table 8S. Effects of 2-year supplementation with vitamin K2 (MK-7, 360 µg daily) or placebo: Biochemical markers of vitamin K status.**

| **Outcome** | **Time** | **Mean levels (95% CI)** | | **Mean change**  **in study groups from baseline (95% CI)** | | **Differences of changes between**  **study groups from baseline (95% CI)** | |
| --- | --- | --- | --- | --- | --- | --- | --- |
|  |  | **Vitamin K** | **Placebo** | **Vitamin K** | **Placebo** |  | **P - value** |
| **S-vitamin K1 (nmol/L)** | Baseline | 0.59 (0.46;0.71)  (n=123) | |  |  |  |  |
|  | 1 year | 0.63 (0.43;0.82) (n=48) | 0.53 (0.33;0.73) (n=47) | 0.04 (-0.18;0.26) | -0.06 (-0.28;0.16) | 0.10 (-0.18;0.37) | 0.49 |
|  | 2 year | 0.73 (0.49;0.98) (n=31) | 0.69 (0.46;0.92) (n=34) | 0.15 (-0.11;0.41) | 0.11 (-0.15;0.36) | 0.04 (-0.29;0.37) | 0.81 |
| **S-MK-7 (nmol/L)** | Baseline | 0.60 (0.49;0.71)  (n=123) | |  |  |  |  |
|  | 1 year | 19.04 (11.78;26.29) (n=48) | 0.60 (0.45;0.76) (n=47) | 18.44 (11.18;25.69) | 0.00 (-0.14;0.15) | 18.43 (13.09;23.78) | **< 0.001** |
|  | 2 year | 25.76 (16.73;34.79) (n=31) | 0.68 (0.51;0.85) (n=34) | 25.16 (16.13;34.19) | 0.08 (-0.09;0.24) | 24.24 (17.81;30.67) | **< 0.001** |
| **P-total-OC (ng/mL)** | Baseline | 216.3 (171.8;260.8)  (n=123) | |  |  |  |  |
|  | 1 year | 248.5 (183.8;313.2)  (n=49) | 295.8 (229.9;361.7)  (n=47) | 32.2 (-32.2;96.5) | 79.5 (13.9;145.0) | -47.3 (-133.9;39.3) | 0.28 |
|  | 2 year | 327.5 (247.4;407.7)  (n=29) | 445.7 (367.7;523.8)  (n=31) | 111.2 (31.4;191.1) | 229.4 (151.7;307.2) | -118.2 (-225.3;-11.1) | **0.03** |
| **P-dp-ucMGP (pmol/L)** | Baseline | 2 113 (1 924;2 302)  (n=123) | |  |  |  |  |
|  | 1 year | 1 228 (966;1 490) (n=49) | 2 461 (2 195;2 728)  (n=47) | -885 (-1 130;-640) | 348 (99;598) | -1 233 (-1 568;-899) | **< 0.001** |
|  | 2 year | 1 737 (1 427;2 047)  (n=31) | 3 721 (3 422;4 021)  (n=34) | -376 (-671;-81) | 1 609 (1 324;1 893) | -1 985 (-2 382;-1 588) | **< 0.001** |
| **P-PIVKA-II (ng/mL)** | Baseline | 40.7 (34.5;47.0)  (n=122) | |  |  |  |  |
|  | 1 year | 21.7 (12.4;31.1)  (n=49) | 44.2 (34.7;53.8)  (n=47) | -19.0 (-28.8;-9.1) | 3.5 (-6.4;13.5) | -22.5 (-35.4;-9.6) | **0.001** |
|  | 2 year | 24.2 (12.7;35.6)  (n=31) | 51.1 (40.0;62.3)  (n=33) | -16.5 (-28.4;-4.7) | 10.4 (-1.1;21.9) | -27.0 (-42.6;-11.3) | **0.001** |

**Supplemental Table 8S.** Mixed effect model analysis of changes in biochemical markers. Variables are presented as mean and 95% Confidence intervals (CI). Abbreviations:

MK-7, menaquinone 7; S, serum; P, plasma; OC, Osteocalcin; dp-ucMGP, dephosphorylated-uncarboxylated matrix Gla protein; PIVKA-II, Protein Induced by Vitamin K Absence-II.

**Supplemental Table 9S. Effects of 2-year supplementation with vitamin K2 (MK-7, 360 µg daily) or placebo: Biochemical markers of mineral and bone turnover.**

| **Outcome** | **Time** | **Mean levels (95% CI)** | | **Mean change**  **in study groups from baseline (95% CI)** | | **Differences of changes between**  **study groups from baseline (95% CI)** | |
| --- | --- | --- | --- | --- | --- | --- | --- |
|  |  | **Vitamin K** | **Placebo** | **Vitamin K** | **Placebo** |  | **P- value** |
| **P-25-OH-vit D2+3 (nmol/L)** | Baseline | 67.6 (61.7;73.4)  (n=119) | |  |  |  |  |
|  | 1 year | 69.3 (60.9;77.7) (n=48) | 69.4 (60.8;78.0) (n=45) | 1.7 (-6.6;10.0) | 1.8 (-6.6;10.3) | -0.1 (-11.3;11.1) | 0.99 |
|  | 2 year | 70.3 (60.4;80.3) (n=31) | 70.7 (61.0;80.5) (n=33) | 2.8 (-7.18;12.7) | 3.2 (-6.5;12.8) | -0.4 (-13.7;12.8) | 0.95 |
| **S-1,25(OH)2 vit D (pmol/L)** | Baseline | 51.7 (46.5;56.8) (n=123) | |  |  |  |  |
|  | 1 year | 56.1 (48.4;63.9) (n=49) | 51.5 (43.6;59.4) (n=47) | 4.5 (-3.5;12.5) | -0.2 (-8.3;8.0) | 4.63 (-6.0;15.3) | 0.39 |
|  | 2 year | 55.2 (45.8;64.6) (n=31) | 60.4 (51.3;69.5) (n=34) | 3.5 (-6.1;13.2) | 8.8 (-0.5;18.0) | -5.2 (-18.0;7.5) | 0.42 |
| **P-iPTH (pmol/L)** | Baseline | 32.5 (27.4;37.6) (n=122) | |  |  |  |  |
|  | 1 year | 37.0 (29.3;44.7) (n=49) | 41.5 (33.6;49.4) (n=46) | 4.5 (-3.5;12.5) | 9.0 (0.7;17.2) | -4.5 (-15.1;6.2) | 0.41 |
|  | 2 year | 33.4 (24.0;42.8) (n=31) | 42.4 (33.4;51.4) (n=34) | 0.9 (-8.8;10.6) | 9.9 (0.5;19.2) | -8.9 (-21.6;3.7) | 0.17 |
| **P-FGF-23**  **(ng/L)** | Baseline | 10 383 (7 746;13 020)  (n=122) | |  |  |  |  |
|  | 1 year | 9 354 (5 899;12 810) (n=49) | 9 646 (6 142;13 150) (n=47) | -1 029 (-4 014;1 956) | -737 (-3 764;2 290) | -292 (-4 413;3 830) | 0.89 |
|  | 2 year | 9 736 (5 698;13 773) (n=30) | 8777 (4 905;12 650)  (n=34) | -648 (-4 295;2 999) | -1 606 (-5 053;1 841) | 958 (-3 950;5 867) | 0.70 |
| **P-Ionized calcium**  **(mmol/L)** | Baseline | 1.18 (1.17;1.20) (n=121) | |  |  |  |  |
|  | 1 year | 1.19 (1.17;1.21) (n=49) | 1.16 (1.14;1.19) (n=48) | 0.01 (-0.02;0.03) | -0.02 (-0.04;0.00) | 0.03 (-0.00;0.06) | 0.07 |
|  | 2 year | 1.19 (1.17;1.22) (n=31) | 1.17 (1.15;1.20) (n=34) | 0.01 (-0.02;0.04) | -0.01 (-0.04;0.01) | 0.02 (-0.02;0.06) | 0.26 |
| **P-Phosphate (mmol/L)** | Baseline | 1.64 (1.46;1.71) (n=123) | |  |  |  |  |
|  | 1 year | 1.60 (1.48;1.71) (n=49) | 1.65 (1.53;1.77) (n=48) | -0.04 (-0.17;0.08) | 0.01 (-0.11;0.14) | -0.06 (-0.22;0.11) | 0.50 |
|  | 2 year | 1.48 (1.34;1.62) (n=31) | 1.58 (1.45;1.72) (n=34) | -0.16 (-0.30;-0.01) | -0.06 (-0.20;0.09) | -0.10 (-0.30;0.09) | 0.31 |
| **P-Magnesium (mmol/L)** | Baseline | 1.02 (0.99;1.06) (n=123) | |  |  |  |  |
|  | 1 year | 1.01 (0.95;1.06) (n=49) | 1.00 (0.95;1.05) (n=49) | -0.02 (-0.07;0.04) | -0.02 (-0.07;0.03) | 0.00 (-0.07;0.07) | 0.91 |
|  | 2 year | 1.05 (0.98;1.11) (n=31) | 1.00 (0.94;1.06) (n=34) | 0.03 (-0.04;0.09) | -0.02 (-0.08;0.04) | 0.05 (-0.04;0.13) | 0.30 |
| **P-BSAP**  **(µg/L)** | Baseline | 13.3 (11.3;15.3)  (n=123) | |  |  |  |  |
|  | 1 year | 19.9 (16.8;23.0) (n=49) | 18.1 (15.0;21.3) (n=47) | 6.6 (3.3;9.9) | 4.8 (1.5;8.2) | 1.8 (-2.5;6.1) | 0.42 |
|  | 2 year | 30.1 (26.3;33.9) (n=31) | 26.5 (22.9;30.1) (n=34) | 16.8 (12.8;20.7) | 13.2 (9.4;17.0) | 3.6 (-1.6;8.7) | 0.18 |
| **P-CTX-1**  **(ng/L)** | Baseline | 1 845 (1 643;2 047) (n=123) | |  |  |  |  |
|  | 1 year | 2 126 (1 824;2 429) (n=49) | 1 974 (1 666;2 282) (n=47) | 282 (-31;595) | 129 (-189;448) | 152 (-261;567) | 0.47 |
|  | 2 year | 2 095 (1 726;2 464) (n=31) | 2 181 (1 826;2 535) (n=34) | 250 (-127;628) | 336 (-28;700) | -86 (-583;412) | 0.74 |
| **P-P1NP**  **(µg/L)** | Baseline | 313.5 (263.7;363.3) (n=123) | |  |  |  |  |
|  | 1 year | 361.3 (289.9;432.8) (n=49) | 392.3 (319.6;465.1)  (n=47) | 47.8 (-22.0;117.6) | 78.8 (7.7;150.0) | -31.0 (-125.4;63.4) | 0.52 |
|  | 2 year | 327.5 (241.9;413.0) (n=31) | 374.8 (292.3;457.3) (n=34) | 14.0 (-70.2;98.2) | 61.3 (-19.8;142.4) | -47.3 (-159.8;65.2) | 0.41 |

**Supplemental Table 9S.** Mixed effect model analysis of changes in biochemical markers. Variables are presented as mean and 95% Confidence intervals (CI).

Abbreviations: MK-7, menaquinone 7; S, serum; P, plasma; Vit D, vitamin D; iPTH, parathyroid hormone; FGF-23, Fibroblast Growth Factor 23; BSAP, Bone Specific Alkaline Phosphatase; CTX-1, Type I Collagen Cross-Linked C-Telopeptide; P1NP, total Procollagen type 1 N-terminal Propeptide.

**Supplemental Table 10S.** Effects of 2-years of supplementation with vitamin K2 (MK-7, 360 µg daily) or placebo: Abdominal Aortic Calcification (AAC) scores

| **AAC scores** | **Time** | **Mean levels (95% CI)** | | **Mean change in study groups from baseline (95% CI)** | | | | | | **Differences of changes between groups from baseline (95% CI)** | | | **P** | |
| --- | --- | --- | --- | --- | --- | --- | --- | --- | --- | --- | --- | --- | --- | --- |
|  |  | **Vitamin K** | **Placebo** | **Vitamin K** | | | **Placebo** | | |  |  |  |  |  |
| **Unadjusted** | Baseline | 9.4 (8.8 ; 10.0)  (n=118) | | | |  | | |  | |  | | |  |
|  | Year 1 | 10.1 (9.3 ; 10.9)  (n=44) | 9.8 (9.0 ; 10.5)  (n=47) | | 0.69 (-0.0 ; 1.4) | | | 0.4 (-0.2 ; 1.0) | | | 0.3 (-0.6 ; 1.2) | 0.51 | | |
|  | Year 2 | 10.8 (9.7 ; 11.9)  (n=26) | 10.3 (9.4 ; 11.1)  (n=32) | | 1.4 (0.4 ; 2.5) | | | 0.9 (0.2 ; 1.6) | | | 0.5 (-0.7 ; 1.8) | 0.41 | | |
| **Adjusted*** | Baseline | 9.4 (8.8 ; 10.0)  (n=118) | | |  | | |  | | |  |  | | |
|  | Year 1 | 10.1 (9.3 ; 10.9)  (n=44) | 9.8 (9.0 ; 10.5)  (n=47) | | 0.7 (-0.0 ; 1.4) | | | 0.4 (-0.2 ; 1.0) | | | -0.7 (-2.7 ; 1.3) | 0.49 | | |
|  | Year 2 | 10.8 (9.7 ; 11.9)  (n=26) | 10.3 (9.4 ; 11.1)  (n=32) | | 1.4 (0.4 ; 2.5) | | | 0.9 (0.2 ; 1.6) | | | -0.5 (-2.5 ; 1.6) | 0.64 | | |

**Supplemental Table 10S**. * Adjusted for gender and age.

**Supplemental Table 11S.** Effects of 2-years of supplementation with vitamin K2 (MK-7, 360 µg daily) or placebo: Lumbar vertebral fractures

| **Lumbar vertebral fractures** | **Vitamin K**  (n= 61) | **Placebo**  (n=62) | **P**  **between study groups**^a^ |
| --- | --- | --- | --- |
| **Baseline**  Number of participants | 2 | 6 | 0.27 |
| Number of fractures | 2 | 7 | 0.16 |
| **Year 2**  Number of participants | 2 | 6 |  |
|  |  |  | 0.27 |
| Number of fractures | 2 | 7 | 0.16 |

**Supplemental Table 11S**. Variables are presented as numbers of participants having lumbar vertebral compression fractures L1-L4 and total number of lumbar vertebral compression fractures, respectively. ^a^ Fisher’s Exact test (n < 5).

**Supplemental Table 12S.** Effects of 2-years of supplementation with vitamin K2 (MK-7, 360 µg daily) or placebo in participants on chronic dialysis treatment: Clinical outcomes.

|  | **Vitamin K**  (n = 61) | **Placebo**  (n = 62) | **P**  **between study groups**^a^ |
| --- | --- | --- | --- |
| **Fractures**  Number of participants | 3 | 5 | 0.72 |
| Number of fractures | 4 | 7 | 0.53 |
| **Parathyroidectomy** | 2 | 2 | 1.00 |
| **Thromboembolic events**  Number of participants | 12 | 16 | 0.42 |
| Number of events | 19 | 32 | **0.02** |
| **Deaths** | 10 | 11 | 0.85 |

**Supplemental Table 12S.** Variables are presented as numbers of participants experiencing a clinical outcome and total number of clinical outcomes, respectively. ^a^ Fisher’s Exact test (n < 5), Pearson’s Chi-Squared test.

**Supplemental Table 13S.** Data Sharing Statement.

| Will individual participant data be available (including data dictionaries)? | Yes |
| --- | --- |
| What data in particular will be shared? | Individual participant’s deidentified data that underlie the results reported in this article (text, tables, and figures). |
| What other documents will be available? | Study protocol (in Danish), Participants information (in Danish), and Informed consent form (in Danish). |
| When will data be available? | Beginning immediately following publication and ending 36 months following article publication. |
| With whom? | Researchers who provide a methodologically sound proposal. |
| For what types of analyses? | To achieve aims in the approved proposal. |
| By what mechanism will data be available? | Proposals should be directed to [karinschousboe@dadlnet.dk](mailto:karinschousboe@dadlnet.dk). |
